# Co-enrichment of cancer-associated bacterial taxa is correlated with immune cell infiltrates in esophageal tumor tissue

**DOI:** 10.1038/s41598-023-48862-3

**Published:** 2024-01-31

**Authors:** K. L. Greathouse, J. K. Stone, A. J. Vargas, A. Choudhury, R. N. Padgett, J. R. White, A. Jung, C. C. Harris

**Affiliations:** 1 Department of Biology, Baylor University, Waco, TX USA; 2 Nutrition Division, Human Sciences and Design, Baylor University, Waco, TX USA; 3 Center for Cancer Research, National Cancer Institute, Bethesda, MD USA; 4 Eunice Kennedy Shriver National Institute of Child Health and Human Development, National Institutes of Health, Bethesda, MD USA; 5 Harvard T.H. Chan School of Public Health, Harvard University, Boston, MA USA; 6 Resphera Biosciences, LLC, Baltimore, MD USA

**Keywords:** Cancer, Cancer microenvironment, Gastrointestinal cancer, Tumour biomarkers

## Abstract

Esophageal carcinoma (ESCA) is a leading cause of cancer-related death worldwide, and certain oral and intestinal pathogens have been associated with cancer development and progression. We asked if esophageal microbiomes had shared alterations that could provide novel biomarkers for ESCA risk. We extracted DNA from tumor and non-tumor tissue of 212 patients in the NCI-MD case control study and sequenced the 16S rRNA gene (V3-4), with TCGA ESCA RNA-seq (n = 172) and WGS (n = 123) non-human reads used as validation. We identified four taxa, *Campylobacter, Prevotella, Streptococcus,* and *Fusobacterium* as highly enriched in esophageal cancer across all cohorts. Using SparCC, we discovered that *Fusobacterium* and *Prevotella* were also co-enriched across all cohorts. We then analyzed immune cell infiltration to determine if these dysbiotic taxa were associated with immune signatures. Using xCell to obtain predicted immune infiltrates, we identified a depletion of megakaryocyte-erythroid progenitor (MEP) cells in tumors with presence of any of the four taxa, along with enrichment of platelets in tumors with *Campylobactor* or *Fusobacterium*. Taken together, our results suggest that intratumoral presence of these co-occurring bacterial genera may confer tumor promoting immune alterations that allow disease progression in esophageal cancer.

## Introduction

Esophageal carcinoma (ESCA) is a rapidly increasing malignancy, with global rates increasing nearly 50% from 2012 to 2019 (Surveillance, Epidemiology, and End Results (SEER), National Cancer Institute). ESCA is predominantly classified as adenocarcinoma (EAC) and squamous cell carcinoma (ESCC), which show striking disparities. EAC is more common in men, younger patients, and Western countries while ESCC is more common in women, older patients, and African and Asian countries^1,2^. Development of ESCA also varies by histology, with EAC linked to a pro-inflammatory condition called Barrett’s esophagus and ESCC linked to environmental factors, including obesity and smoking, and for both somatic mutations such as *TP53*^3–6^.

These risk factors are also known to play a role in modulating the gastrointestinal microbiome^7^. Several studies have demonstrated community and taxonomic alterations of the esophageal microbiome in ESCA patients^8–10^, showing a transition from Gram-positive dominated to a Gram-negative dominated microbiota before development of EAC. In vivo studies indicate that microbiome changes occur during the development of ESCA that correlate with changes in gene expression in the esophageal epithelium, including multiple microbial sensing pathways (e.g. toll-like receptors) that influence immune signaling and immune cell recruitment patterns^11,12^. These data suggest that alterations within the microbiome, or dysbiosis, may result in chronic inflammation and contribute to the development of ESCA.

To better understand how microbial dysbiosis and its interplay with the immune system contributions to ESCA development, we analyzed the microbiome of three datasets from the National Cancer Institute-Maryland (NCI-MD) case control study, The Cancer Genome Atlas (TCGA) RNA sequencing (RNA-seq) and whole genome sequencing (WGS) datasets. We compared taxonomic abundance between non-tumor adjacent and tumor tissues and identified four genera, *Campylobacter*, *Fusobacterium, Prevotella*, and *Streptococcus*, co-enriched in each dataset. Further examination of clinicopathological factors such as gender, race, smoking status, and histology revealed no association with the above four taxa. However, taxa association with immune cell abundance suggested platelet differentiation was increased in tumors with high taxa abundance. These data suggest certain opportunistic pathogenic taxa may promote esophageal cancer development by altering the immune microenvironment.

## Results

**Figure 1:**
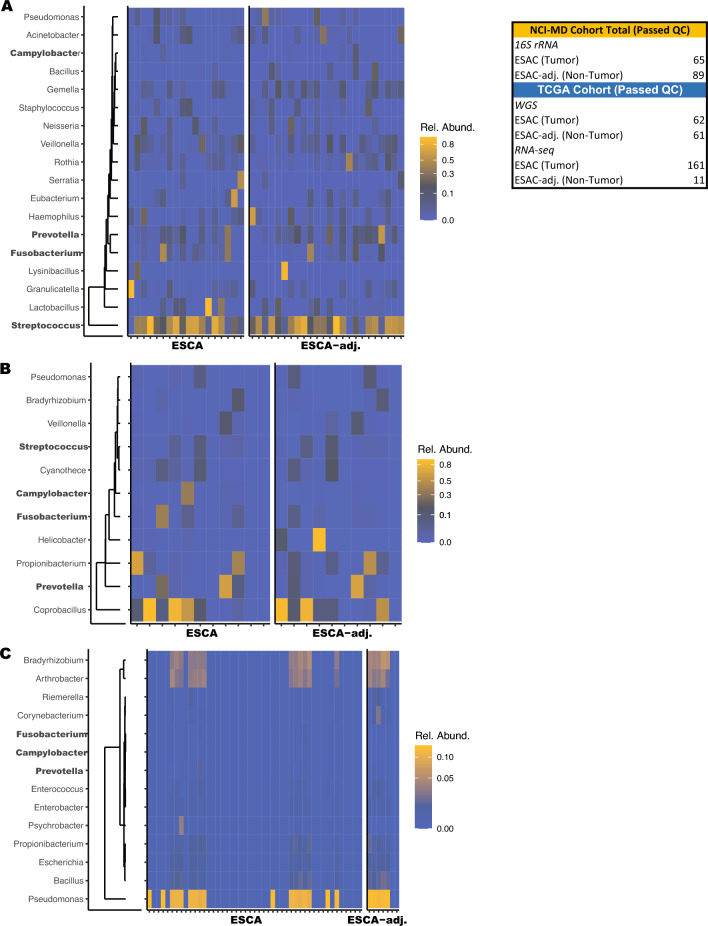
Identification of microbial signatures in esophageal cancer. (**A**) Bacterial abundance within the NCI-MD case control study calculated from 16S V3-4 amplification. ESCA adj. indicates non-tumor adjacent tissue. (Panel) Total number of patients used in this study from three cohorts: NCI-MD case control study, TCGA RNA-seq, and TCGA whole genome seq (WGS). (**B**) Bacterial abundance within TCGA WGS, determined by quantification of non-human aligned reads. (**C**) Bacterial abundance within TCGA RNA-seq, determined by quantification of non-human aligned reads. A 1% cutoff was applied to all taxa as the minimal average (across samples) for plotting.

To better understand the microbial and immune system contributions to ESCA development, we comprehensively evaluated the esophageal tissue microbiome and inferred immune cell infiltration in patients with ESCA in two cohorts, NCI-MD and TCGA, with latter divided into WGS and RNA-seq, which results in three datasets. We extracted DNA from patients enrolled in the NCI-MD case control study from the Baltimore, Maryland area (118 non-tumor tissues, 94 tumors; 45 NT-T pairs) for 16S V3-4 sequencing as previously described^13^. Briefly, sequence reads were filtered for length (> 200bp) and max error rate (0.5%), and submitted for high-resolution taxonomic assignment (Resphera Insight) to assess taxa abundance. After QC, 154 samples were used for analysis. TCGA ESCA RNA-seq data (11 non-tumor tissue, 162 tumors; 11 NT-T pairs) and whole genome sequencing (WGS) (61 non-tumor tissue, 62 tumors; 61 NT-T pairs) data were downloaded (GDC Data Portal, NCI) as validation cohorts (Fig. 1A, Tables S1-S3). Stringent quality control measures were applied on both data sets (Methods).

First, we sought to determine if any differences existed in the microbiome between ESCA tumor and non-tumor adjacent tissues. No differences in alpha or beta diversity were seen for the NCI-MD cohort, however alpha diversity decreased significantly in TCGA RNA-seq tumor samples but increased significantly in TCGA WGS tumor samples (Figure S1). Because tobacco smoking is a key risk-factor for developing esophageal cancer^14^, we asked if smoking status or other key ESCA risk factors were associated with alpha diversity. Interestingly, none of these clinicopathological factors (gender, histology, race, smoking status, or stage) showed significant difference in taxa abundance across all three cohorts (Figure S2).

**Figure 2:**
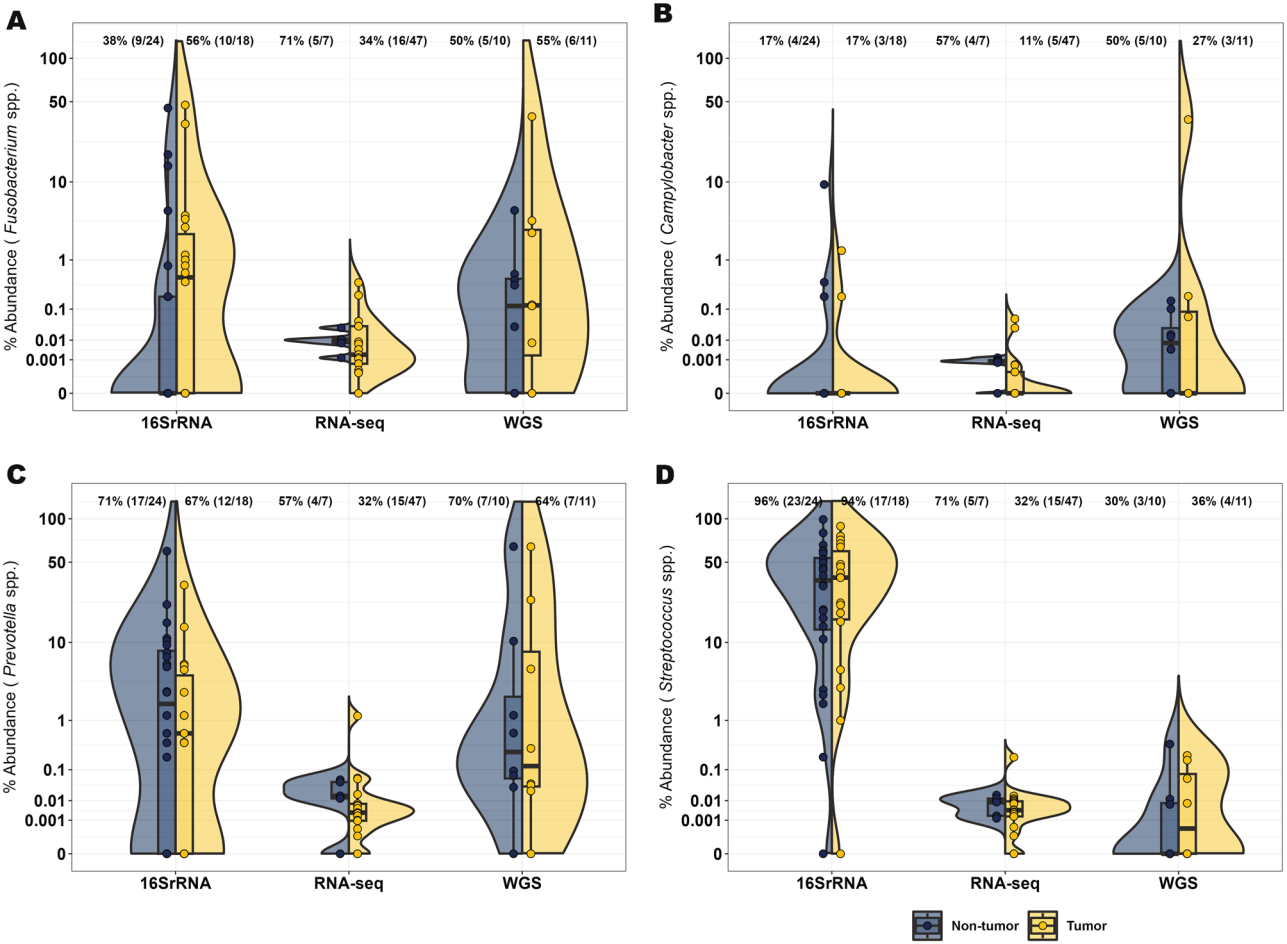
Four taxa are enriched in esophageal cancer across cohorts. Abundance of (**A**) *Fusobacterium,* (**B**) *Campylobacter*, (**C**) *Prevotella,* and (**D**) *Streptococcus* within NCI-MD case control study calculated from 16S V3-4 amplification. Abundance of the above four taxa within TCGA WGS and RNA-seq, determined by quantification of non-human aligned reads. Violin plots indicate relative abundance of each of the four taxa; % is the number of tumors with taxa present (ratio is number of samples with taxa present over number of total samples). Co-association was determined by statistical concordance analysis (Methods).*Statistical concordance analysis is located in Table S4.

Examination of the most abundant taxa in the NCI-MD cohort, independent of tissue type, identified *Streptococcus, Pseudomonas*, *Prevotella, Veillonella, Lactobacillus*, *Stenotrophomonas, Fusobacterium,* and *Acinetobacter*. One (*Pseudomonas*) and six (*Streptococcus, Pseudomonas*, *Prevotella, Veillonella, Lactobacillus*, and *Fusobacterium*) of these taxa were also highly abundant in TCGA RNA-seq and WGS cohorts, respectively (Fig. 1). We then performed a statistical concordance analysis (Methods, Table S4), which identified four common taxa across all cohorts: *Campylobacter*, *Fusobacterium, Prevotella*, and *Streptococcus* as enriched in ESCA (Fig. 2). All four taxa were enriched in tumors in at least two of three cohorts (Table S4). Additionally, given that *TP53* is one of the most frequently mutated genes in ESCA^4,6^, we investigated *TP53* mutation status in the TCGA cohort (WGS and RNA-seq) and found no relationship with abundance of these four taxa (Figure S3).

Comparison of EAC versus ESCC revealed no significant differences in abundance of the four above taxa in any cohort (Figure S4) and expansion to include all taxa returned no differences after Benjamini–Hochberg correction. To determine if any relationship existed with microbial function, apart from community structure, we investigated the inferred metabolic profile of the ESCA microbiome using PICRUSt, but did not identify any associations with ESCA between tumor vs non-tumor tissue, overall or stratified by the four taxa (data not shown).

**Figure 3:**
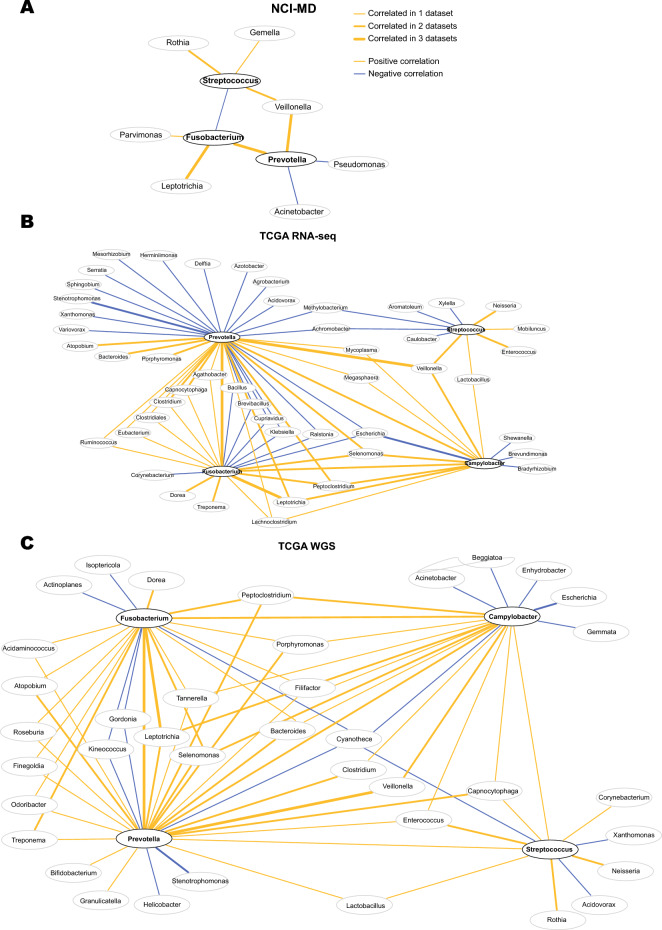
*Fusobacterium* and *Prevotella* are consistently co-associated across cohorts. (**A**) Taxa co-enrichment networks within the NCI-MD case control study. Taxa abundance was permuted through SparCC with 100 iterations, and correlation coefficients were filtered for X < − 0.2 and X > 0.2. Gold edges indicate positive coefficients demonstrating co-enrichment while blue edges indicate negative coefficients demonstrating exclusion. Edge thickness represents normalized coefficient values. (**B**) Taxa co-enrichment networks within TCGA RNA-seq. (**C**) Taxa co-enrichment networks within TCGA WGS. Networks for (**B**) and (**C**) were filtered for correlation coefficients X < − 0.3 and X > 0.3, otherwise networks were constructed as described for (**A**).

Having observed *Fusobacterium* was one of the most enriched taxa in ESCA tissue, we assessed whether this genus is co-abundant with specific taxa in ESCA. Specifically, *Prevotella*, and *Streptococcus* are often found in oral biofilms alongside *Fusobacterium nucleatum* where the two genera rely on *F. nucleatum* binding to salivary protein anchors (e.g. Statherin) and sharing nutrients to grow^15^. Furthermore, *Fusobacterium* and *Prevotella* have been previously described as co-enriched in ESCA^9,16^; therefore we asked if any of our four enriched taxa (*Campylobacter, Fusobacterium, Prevotella*, and *Streptococcus*) were co-enriched in the same tumors or were associated with other taxa. As most microbiome data is often sparse, correlation coefficients calculated by Pearson or Spearman methods are prone to spurious and false-positive relationships^17^ within such community networks^18–20^, so we used SparCC to compensate for the sparsity inherent to 16S-based studies^21^. We calculated co-enrichment for each of the three datasets. Overall, TCGA (RNA-seq and WGS) results were highly concordant (Table S5), and showed a consistent co-enrichment of certain taxa, with *Fusobacterium* and *Prevotella* co-enriched across all cohorts (Fig. 3A–C, Figure S5). These two taxa were also enriched with *Leptotrichia* and *Veillonella*, consistent with prior reports in colorectal cancer^22,23^. The NCI-MD cohort beta diversity suggested that *Streptococcus-*enriched samples were divergent from those with *Campylobacter, Fusobacterium,* and *Prevotella* (Figure S1E). We confirmed a negative co-enrichment between *Streptococcus* and *Campylobacter* and *Fusobacterium* in this cohort (Fig. 3A). Given this negative association, we used our TCGA WGS data to investigate species-level differences, and found a significant enrichment in *S. oralis, F. nucleatum, P. denticola and P. intermedia* in tumors as compared to non-tumors (Figure S6). Regardless of cohort, we found these four taxa were negatively associated with *Acinetobacter, Brevundimonas, Klebsiella, Pseudomonas,* and *Xanthomonas* (Fig. 3A–C). These data indicate that co-occurrence of *Fusobacterium* and *Prevotella* are a common feature of ESCA and may be important in ESCA pathology.

**Figure 4:**
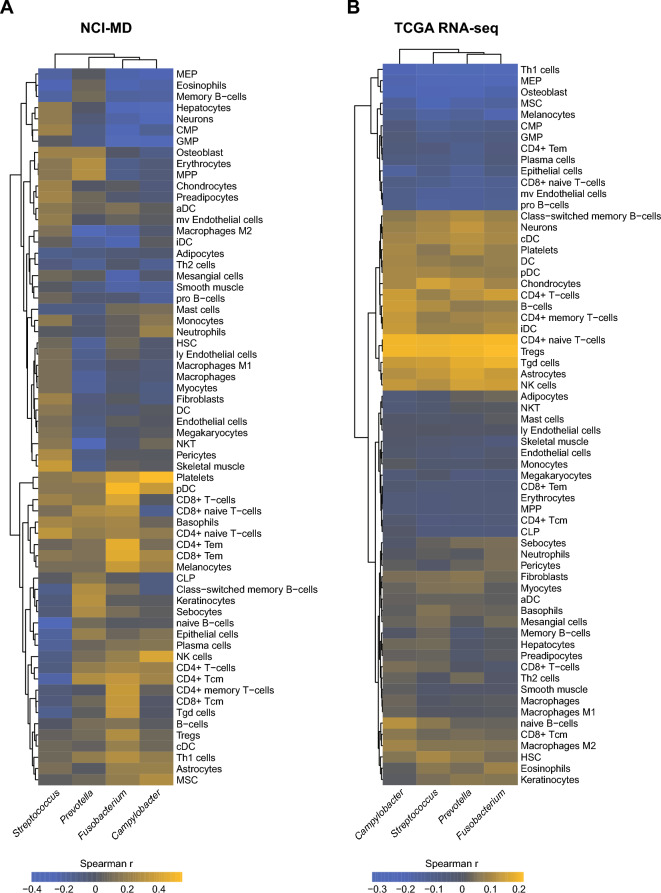
Megakaryocyte–erythroid progenitor cells are depleted in tumors with high carriage of ESCA-enriched taxa. (**A**) RNA-sequencing was performed on NCI-MD patients (n = 23; non-tumor = 13, tumor = 10) and samples were analyzed for predicted cell infiltration using xCell (citation). Cell infiltrates and taxa abundance were correlated using Spearman’s coefficient. (**B**) Correlation of xCell predicted cell infiltration in TCGA RNA-seq patients with taxa abundance. *Statistical significance analysis in Fig. S7-8.

Since *Fusobacterium* spp. have demonstrated effects on gene expression changes within tumor epithelial and immune cells, we predicted immune cell infiltration from NCI-MD and TCGA RNA-seq data using the deconvolution algorithm xCell^24^, and then compared their abundances with our four co-enriched taxa. We identified a depletion of megakaryocyte-erythroid progenitor (MEP) cells in tumors with presence of any of the four bacteria (Fig. 4A,B)*,* which was significant in the RNA-seq TCGA dataset (*p* < 0.001) (Figure S7-S8). Furthermore, we found a modest enrichment of platelets in tumors with *Campylobactor* or *Fusobacterium* (*p* < 0.06) (Fig. 4A,B, Figure S9). These data suggest that intratumoral presence of these bacterial genera results in loss of MEPs by promoting their terminal differentiation to platelets.

## Discussion

Globally rising rates of ESCA suggest that potentially novel drivers are partially responsible, however identification of these factors remains a significant problem in diagnosis and treatment. In this study we asked if the microbiome, a known driver of various GI malignancies^25^, was altered in ESCA in comparison to non-tumor adjacent esophageal tissue. We found enrichment of the taxa *Campylobacter, Fusobacterium, Prevotella,* and *Streptococcus* in tumor tissue. These findings are consistent with other studies examining the ESCA microbiome^8^, although our study is the first to report the co-enrichment of all four taxa in the same cohorts. Additionally, as these taxa exist within a community, abundance changes in one taxon are associated with changes in others, and we found consistent co-enrichment networks with the above four taxa common across cohorts, including associations of *Fusobacterium, Prevotella, Leptotrichia,* and *Veillonella*. Interestingly, these taxa may also invoke terminal differentiation of MEPs into platelets within the tumor microenvironment. *Streptococcus* spp. induce platelet activation and secretion through FcγRIIA signaling^26^. Lipopolysaccharide, the major outer membrane component in Gram-negative microbes such as *Campylobacter*, *Fusobacterium*, and *Prevotella*, also activate platelets through TLR4 signaling and induces proinflammatory cytokine secretion^26–28^. This suggests that the intratumoral presence of these bacteria may result in MEP to platelet differentiation. Expanded platelet counts are known to play a role in esophageal cancer development and metastasis^29^. Platelet-derived growth factor A (PDGFA) increases proliferation and invasion of multiple cancer types and high expression is a poor prognostic factor in ESCA^30^. Platelets may also promote metastasis through increasing interactions between primary tumor and endothelial cells, as occurs in colorectal cancer^30,31^, suggesting these taxa may contribute to increased platelet counts and poorer ESCA prognosis.

A common finding among several cancers of the GI tract, from oral squamous cell carcinoma to colorectal cancer, is the enrichment of *Fusobacterium spp.*, including *Fusobacterium nucleatum*^32^. This species in particular has been shown in many studies to be not only enriched in the tumor but also in adjacent biofilms^33^. *Fusobacterium* is known to be an important bridging species during dysbiosis development, by colonizing early and allowing other taxa, including *Streptococcus*, to form links to additional species to create and establish biofilms^32^. Biofilms are seen in multiple cancer types, including oral and colon, and create opportunities for the growth of more virulent or pathogenic strains of certain Gram-negative microbes^11,33^. This increased virulence allows for the attachment of *Fusobacteria* and other oral and GI enriched microbes to adhere to sugar molecules and proteins on the surface of epithelial and immune cells^34,35^. These attachments create opportunities for invasion of *Fusobacterium* and *Porphyromonas spp.* to invade epithelial cells, promote inflammatory cell signals, and enhance epithelial cell motility to promote extraversion and metastasis^36^. However, it is important to recognize that more than one pathogen, like *F. nucleatum*, is likely required to initiate or promote cancer development or metastasis^37^. Thus, the coordinate efforts between bridging species such as *F. nucleatum*, and others like those we found co-enriched in ESCA tumors, can create the opportunity for other pathogens to work together to promote cancer.

These findings suggest that therapeutic anti-cancer strategies targeting one genus or species are likely to fail as other associated taxa may offset the loss of those taxa. Instead, it is likely a multi-targeted strategy, based on presence of intratumoral taxa, for modulating microbial dysbiosis is required to improve treatment and patient outcome. Further research is needed, however, to better understand the mechanisms driving enrichment of these taxa and immune cells in ESCA and other cancers.

## Methods

### University of Maryland (UMD) esophageal samples: collection and DNA extraction

DNA was extracted from 212 esophageal tissue samples, one plain water control (Mo Bio Laboratories, Inc, Carlsbad, CA, USA), one water control (Mo Bio Laboratories, Inc, Carlsbad, CA, USA) that was carried through the DNA extraction process, and one mock community (BEI resources, Manassas, VA, USA). Esophageal tissue was collected at the University of Maryland Medical Center (Baltimore, MD, USA) under an IRB-approved collection protocol (OH98CN027/ FWA00005897, National Institutes of Health Institutional Review Board) where all surgical subjects gave informed, written consent prior to collection, and all procedures were performed in accordance with the Declaration of Helsinki^31,32^. The study was approved by National Institutes of Health Institutional Review Board. Samples were flash-frozen and stored at − 80 °C until DNA extraction. Tumor stage, histology, and Barrett’s Esophagus status were determined from the pathology report. All work areas were cleaned with 70% ethanol and 10% bleach prior to DNA extraction. DNA extraction was carried out by lysing the microbes in fresh frozen tissue samples using Yeast Cell Lysis Buffer (Epicentre, Madison, WI, USA) and bead beating. Proteinase K and RNAse A were added to the samples to remove proteins and RNA, and to enrich for DNA. Samples were processed through gDNA column (Invitrogen, Carlsbad, CA, USA) and eluted in certified DNA- and RNA-free water (Mo Bio Laboratories, Inc, Carlsbad, CA, USA)^25^.

### University of Maryland (UMD) esophageal samples: PCR amplification and MiSeq sequencing

PCR amplification of the V3-4 region of the 16S rRNA gene in each sample was completed at three different dilutions of genomic DNA (1×, 10× and 100×), and the PCR reaction with the highest yield was carried forth to sequencing as previously described^27^. This process is designed to overcome the inhibitory effect of a large amount of human DNA in esophageal tissue samples. Paired-end DNA sequencing of the amplicons from all samples and both variable regions were completed in the same run on a MiSeq machine (M04141, Illumina, San Diego, CA, USA) using the 2 × 300 base pair chemistry (Reagent barcode: MS3917443-600V3) and 50 unique sample barcodes. All dilutions, PCR amplification and sequencing were completed at the University of Minnesota Genomics Center (Minneapolis, MN, USA).

### 16S rRNA Sequence analysis

Raw read pairs from the MiSeq platform were trimmed for quality using Trimmomatic^38^ with a target final error rate of 0.5%, and merged into consensus fragments with FLASH^39^. High-quality unmerged forward reads (≥ 200bp after trimming) were also included for downstream analysis to increase sample coverage. PhiX spike-in fragments were detected using BLASTN^40^ and removed. Sequences associated with PCR chimeras were identified using UCLUST^41^ and filtered. Human genome contaminant identification was performed by aligning sequences against hg19 using Bowtie2^42^, and mitochondria and chloroplast removal utilized assignments by the RDP classifier^43^. Of the 212 original samples extracted and sequenced, 154 remained after performing the above filtering and contamination steps. Barrett’s esophagus samples were removed from downstream analysis due to low quality reads, leaving only 5 samples remaining, which was too few for analysis. Passing 16S rRNA gene sequences were assigned a high-resolution taxonomic lineage using Resphera Insight, a custom 16S rRNA bioinformatics pipeline that utilizes both the SILVA and Greengenes databases for alignment^33,44^. This analysis utilized a data processing, checking and exploration 9-step process as described in https://greathouselab.github.io/esoph-micro-cancer-workflow/data_processing_nci_umd.html.

To filter out contaminant organisms associated with DNA extraction kit reagents and other sources, we first reviewed negative controls / blank samples prepared with original tissue samples, and developed a set of dominant *indicator contaminant* species including *Bradyrhizobium spp., Propionibacterium_acnes, Agrobacterium_tumefaciens, Delftia spp. and Ralstonia spp*. We then performed a correlation analysis between all species/OTUs and these indicator species. Any species/OTU with a nonparametric Spearman correlation ≥ 0.25 was then considered to be a contaminant and was removed; however 10 species/OTUs with known human body site associations were retained including: *Faecalibacterium prausnitzii, Prevotella copri, Collinsella aerofaciens, Lactobacillus rhamnosus, Prevotella nigrescens, Prevotella disiens, and Finegoldia magna*. To filter very low frequency contaminants, we further removed all members associated with a set of genera known to be contaminants from prior literature^45^*: Bradyrhizobium, Ralstonia, Delftia, Agrobacterium, Janthinobacterium, Halomonas, Methylobacterium, Aquamicrobium, Diaphorobacter, Herbaspirillum, and Variovorax*. After contaminant removal, samples were normalized through rarefaction to 500 sequences per sample. Alpha and beta-diversity analysis performed with QIIME^46^.

### Processing of The Cancer Genome Atlas (TCGA) samples

RNA-seq and WGS bam files reflecting cancer and non-cancer samples from esophageal carcinoma patients available from TCGA were identified using the Genomic Data Commons (GDC) portal and downloaded using the GDC data transfer client (http://portal.gdc.cancer.gov/; Link: https://nam02.safelinks.protection.outlook.com/?url=https%3A%2F%2Fwww.ncbi.nlm.nih.gov%2Fgeo%2Fquery%2Facc.cgi%3Facc%3DGSE234304&data=05%7C01%7CLeigh_Greathouse%40baylor.edu%7C9b71dac5157d46f4d99008dbae42459d%7C22d2fb35256a459bbcf4dc23d42dc0a4%7C0%7C0%7C638295371882327356%7CUnknown%7CTWFpbGZsb3d8eyJWIjoiMC4wLjAwMDAiLCJQIjoiV2luMzIiLCJBTiI6Ik1haWwiLCJXVCI6Mn0%3D%7C3000%7C%7C%7C&sdata=GBWGhA7NtppWJL7UcfC5M0x3JM%2B3Z9Tei7nL1fzT6eM%3D&reserved=0 Token (password)—ctchamkodpurjqf). Barrett’s esophagus status, tumor stage, gender, race and survival information were also retrieved when available from the GDC.

### Quality control and identification of microbial DNA

Unmapped sequences from the raw RNA-seq and WGS bam files were converted to FASTQ format using Samtools^47^ and trimmed for quality with Trimmomatic^38^ to remove error-prone reads. Additionally, in order to remove unmapped spliced transcripts and other poorly aligning sequences, we performed a local alignment to the human reference (hg19) using Bowtie2^42^. Clean sequences passing all filters were assigned to a taxonomic lineage using Pathoscope (v1.0)^48,49^. To filter out contaminant organisms associated with DNA extraction kit reagents and other laboratory sources, we developed a set of 10 dominant indicator contaminant species including members of *Bradyrhizobium, Propionibacterium, Pseudomonas,* and Arthrobacter. We then performed analysis between all species/OTUs and these indicator species across WGS tumor, WGS normal and RNA-seq samples. Any species detected in at least 58 of RNA-seq samples, or 55 of WGS tumor or 55 of WGS normal samples was often found to show a strong Spearman correlation with one or more of the indicator contaminant species, and were thus assigned putative contaminant status. We further removed all species associated with a set of higher taxa known to be contaminants from published literature^45^ or that were also highly recurrent across most samples including members of Pseudomonadales, Comamonadaceae, Rhizobiales, Burkholderiales, Paenibacillaceae, Propionibacterium acnes, Escherichia, and Bacillaceae.

### Integration of 16S rRNA and TCGA microbial profiles

In order to provide a direct comparison between the 16S rRNA and TCGA WGS/RNA-seq microbial profiles, we first performed a concordance study at the species level across all technologies. Manual examination of the WGS/RNA-seq and 16S rRNA data revealed that some species in WGS previously determined to be contaminant were more likely to reflect true oral and upper respiratory tract species (such as *Rothia mucilaginosa* and *Streptococcus mitis*). Therefore, we revisited the contaminant removal process for our data integration of 16S rRNA WGS / RNA-seq data, and rescued species that were present in the 16S rRNA contaminant-free dataset, or those reported in a second esophageal tissue 16S rRNA study by Gall et al.^50^. This effort confirmed consistent taxonomic profiles for joint interpretation across genomic data types.

### Inferred microbial metabolism

The input files were a FASTA file of representative sequences and a BIOM table of the abundance of each ASV across each sample from the NCI-MD cohort. The steps of the pipeline used were (1) sequence placement, (2) hidden-state prediction of genomes, (3) metagenome prediction, and (4) pathway-level predictions. The following pipeline was followed to perform this analysis: https://github.com/picrust/picrust2/wiki/Full-pipeline-script

### Statistical methods

Statistical comparisons were performed in R (cran.r-project.org). To establish associations of specific microbial members with tumor status, we utilized Generalized linear fixed effects models (GLMs) and Generalized linear mixed effects models (GLMMs) in which patient membership was considered a fixed effect, or random effect, respectively. The Mann–Whitney test for differential abundance was applied per each genomic data type independent as a supplement to GLM analyses. Fisher’s exact test was applied to evaluate differential frequencies of positive vs negative status for each microbial member in the integrated analysis. *Generalized linear models* – taxon % abundance modeled by Tumor / Normal status (fixed effect) and Patient ID (fixed effect) (stratified by genomic data type). *Generalized linear mixed effects models* – taxon % abundance modeled by Tumor / Normal status (fixed effect) and Patient ID (random effect) (stratified by genomic data type). Fisher’s exact test for positive status (stratified by genomic data type). Comparisons to adjust for the blood-derived normal samples in TCGA were also applied. *Creation of heatmaps* – cases were subset to only those without Barrett’s Esophagus with EAC. OTUs below a minimum threshold of average relative abundance were removed (e.g., an average of 1% relative abundance). The heatmaps plot the individual tissues along the X-axis and the genus abundance along the Y-axis after filtering for the minimum threshold of relative abundance, with cell shading based on the individual genus relative abundances. The scale of shading was adjusted for each data source due to differences in average relative abundance. Hierarchical clustering of tissues and genera was performed using the hclust(.) function in R with default method complete-linkage. Code to replicate the heatmaps is available in our code repository under the file “Fig. 1_heatmaps.R”. Heatmaps were generated using the pheatmap package (v1.0.12). All analyses for the main figures are located at https://github.com/GreathouseLab/esoph-micro-cancer-workflow

### RNA-sequencing and immune infiltration

Total RNA was extracted from fresh-frozen esophageal tissues using TRIzol. RNA quality was validated by Agilent TapeStation and samples with RIN value ≥ 7.0 were selected for sequencing. Samples were sequenced on the DNBseq platform with 2 × 100 bp paired-end sequencing. Reads were aligned to the human genome (hg38) using HISAT and bowtie2^24,42,51^. xCell was used to predict immune cell infiltration in each sample^24^ and predicted infiltrates were correlated with microbial abundance by the Spearman method.

### Microbial co-abundance networks

For each cohort, taxa co-occurrence was calculated using SparCC with 100 iterations and default correlation method (not Pearson or Spearman)^18^. Correlation coefficients were filtered for X < − 0.2 and X > 0.2 (NCI-MD) or X < − 0.3 and X > 0.3 (TCGA). Networks were generated using Cytoscape v3.9.1.

### Supplementary Information

Supplementary Information.

## Data Availability

All de-identified data and code used to conduct analyses and generate figures for this manuscript are available from TCGA or at https://github.com/GreathouseLab/esoph-micro-cancer-workflow. All sequences generated during this study are deposited under the GEO accession #GSE234304. Any protocols will be made available at the request of the researcher by contacting Dr. Leigh Greathouse.
