# Supplementary material for: Co-enrichment of cancer-associated bacterial taxa is correlated with immune cell infiltrates in esophageal tumor tissue
Source: Sci Rep. 2024 Jan 31;14:2574. doi: 10.1038/s41598-023-48862-3 (PMC10831118; doi:10.1038/s41598-023-48862-3)
Supplement: Supplementary file 1 — Supplementary Information. [file 41598_2023_48862_MOESM1_ESM.pdf]

SUPPLEMENTARY MATERIALS

Title: Co-enrichment of cancer-associated bacterial taxa is correlated with immune cell infiltrates in esophageal tumor tissue

Authors: Greathouse, KL*^[1,2], Stone, JK*[3], Vargas, AJ[4], Choudhury, A[1], Padgett, N[5], White, JR[6], Jung, A[1], Harris, CC[3]

Author Affiliations:
1. Department of Biology, Baylor University, Waco, TX
2. Nutrition Division, Human Sciences and Design, Baylor University, Waco, TX
3. Center for Cancer Research, National Cancer Institute, Bethesda, MD
4. *Eunice Kennedy Shriver* National Institute of Child Health and Human Development, National Institutes of Health, Bethesda, MD
5. Harvard T.H. Chan School of Public Health, Harvard University, Boston, MA
6. Resphera Biosciences, LLC, Baltimore, MD

*Co-first authors
^Corresponding author

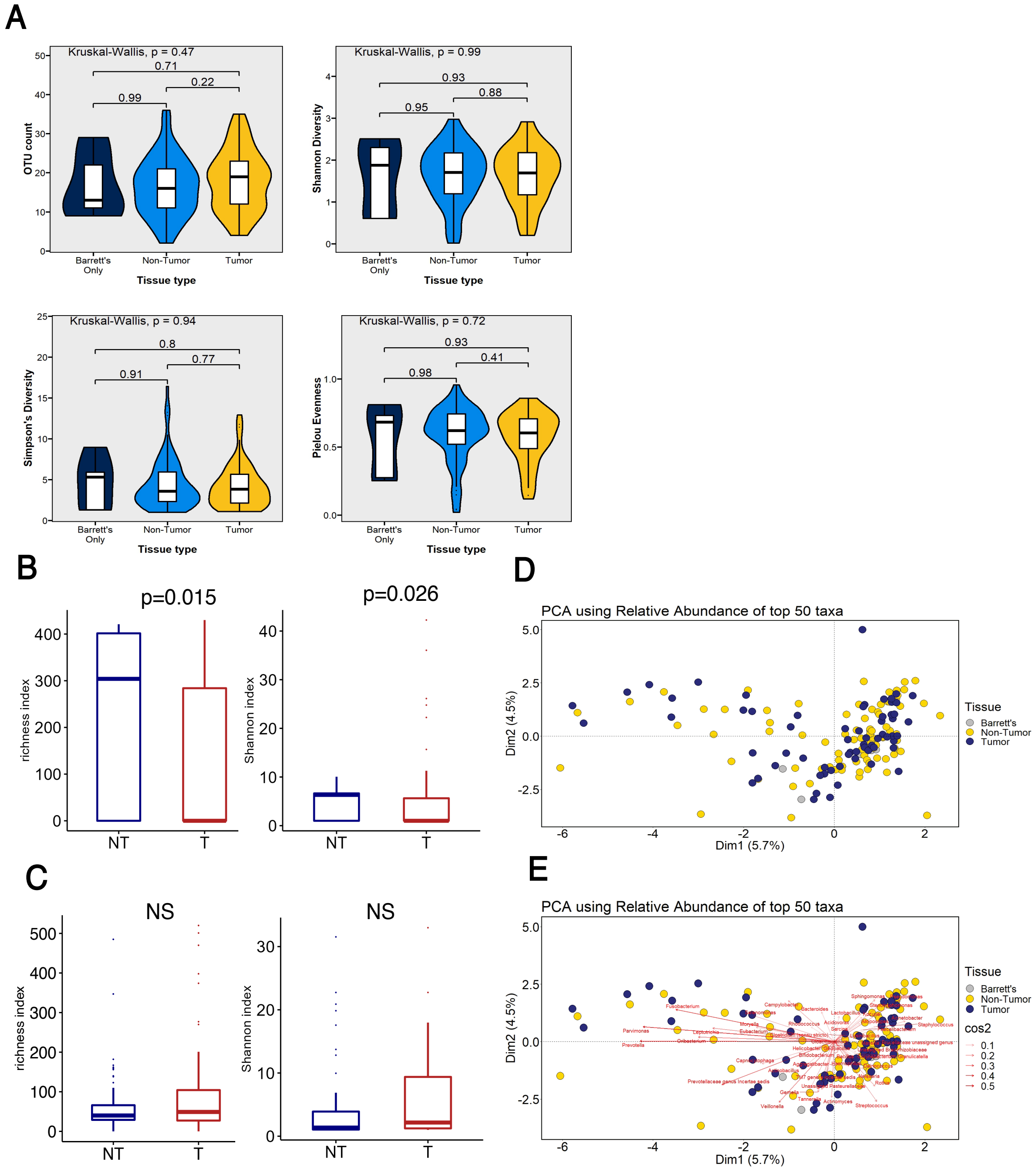

Fig. S1: Alpha and beta diversity are variable across cohorts.

Alpha diversity within the NCI-MD and TCGA cohorts. **A-C** Violin or box plots indicate median values with upper and lower quartiles. Significance determined by Kruskal-Wallis test. **C** Alpha diversity in **B** TCGA RNA-seq and **C** WGS cohorts. Boxplots indicate median values with upper and lower quartiles. Significance determined by Wilcoxon test. **D-E** Beta diversity within the NCI-MD case control study determined by Bray-Curtis. **E** Compositional PCoA biplot of beta diversity with arrows signifying highly ranked taxonomic features contributing the most difference. Significance determined by PERMANOVA test. For all graphs, n.s. not significant, * *p* < 0.05.

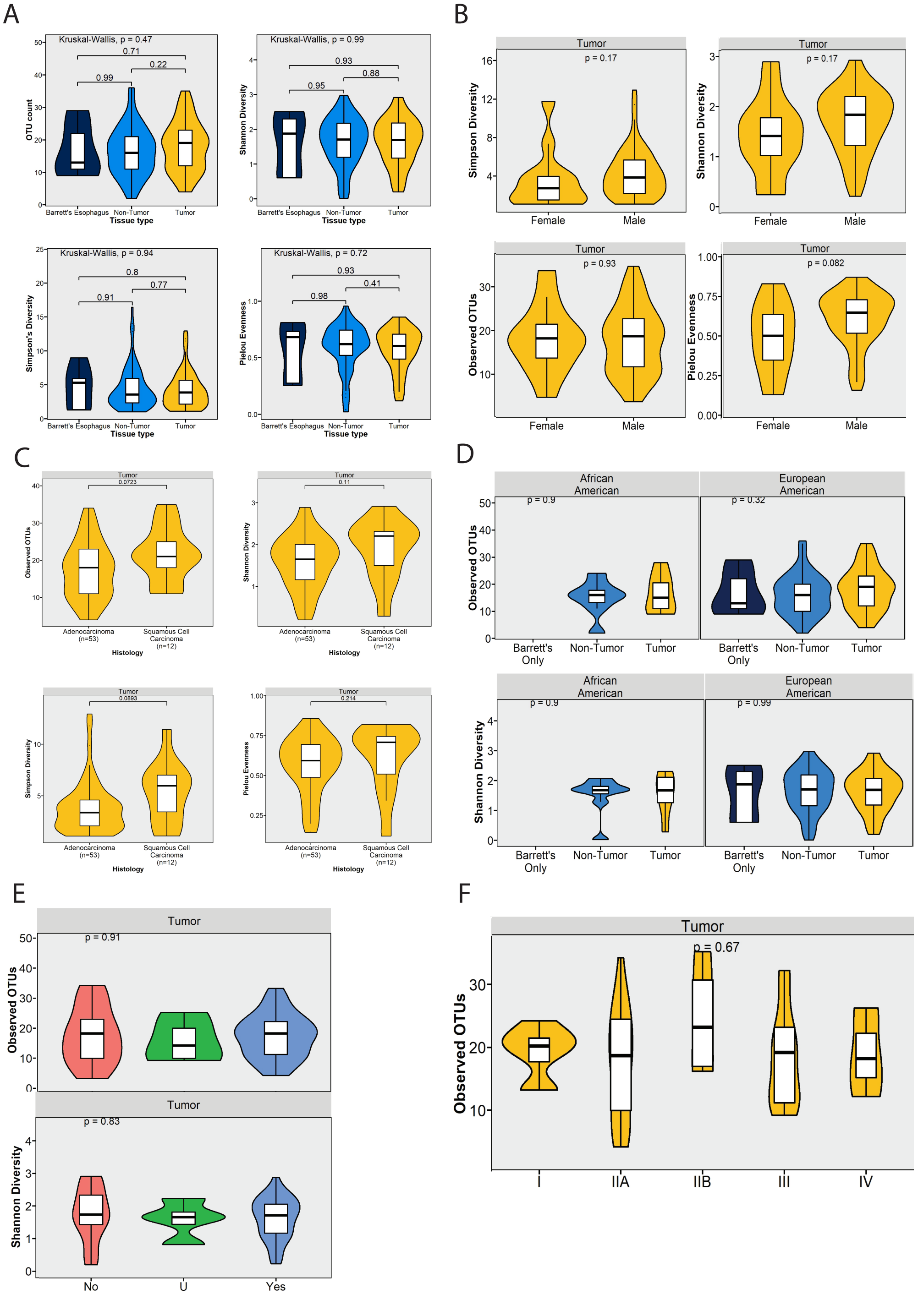

Fig. S2: Taxa abundance are not associated with risk factors for esophageal cancer in NCIMD cohort. Taxa abundance in NCI-MD case control study for **A** tissue type, **B** gender, **C** histology, **D** race, **E** smoking status, and **F** stage. For all graphs, violin plots indicate median values with upper and lower quartiles. Significance was determined by Kruskal-Wallis test n.s. not significant, * $p < 0.05$, ** $p < 0.01$, *** $p < 0.001$.

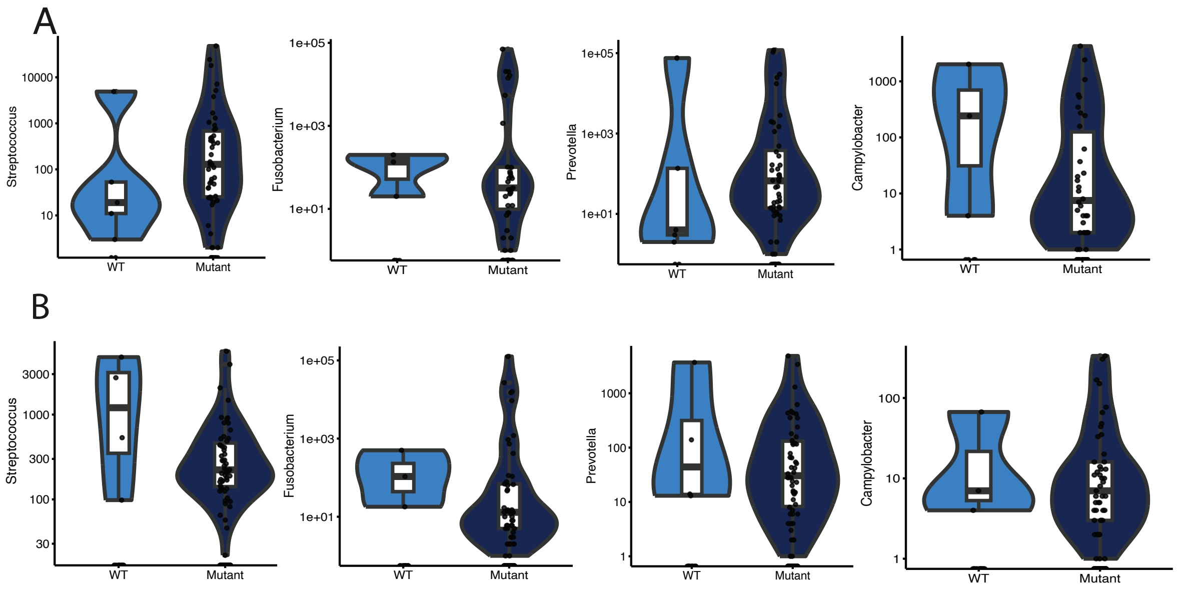

Fig. S3 - Taxa abundance are not associated with *TP53* mutation status in ESCA TCGA WGS and RNA-seq cohort. Taxa abundance TCGA WGS study for TP53 mutation status in **A** WGS and **B** RNA-seq dataset. For all graphs, violin plots indicate median values with upper and lower quartiles. Significance was determined by Kruskal-Wallis test n.s. not significant, * $p < 0.05$, ** $p < 0.01$, *** $p < 0.001$.

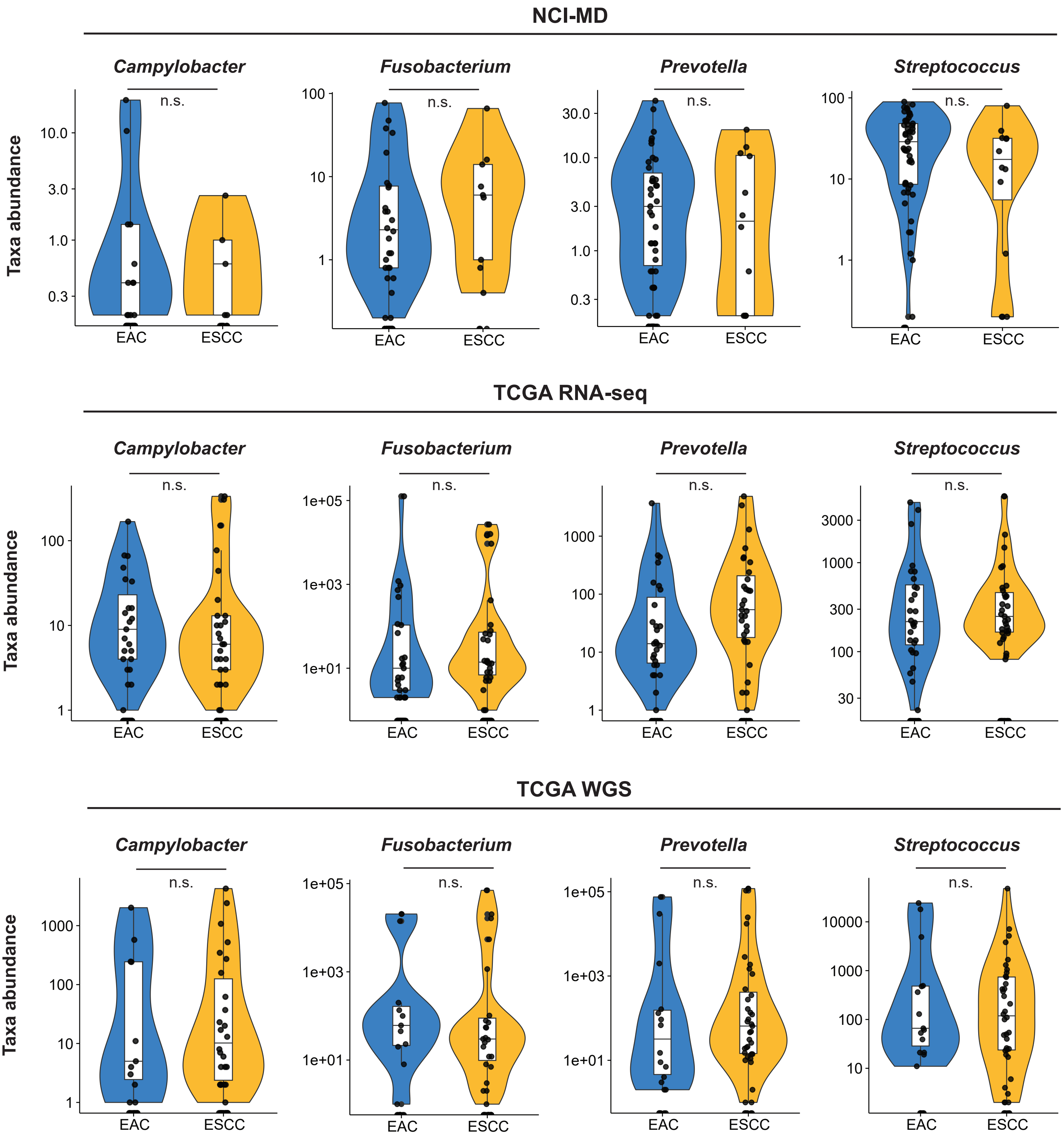

Fig. S4. Histological subtype is not associated with abundance of enriched taxa. For the RNA-seq (top panel) and WGS (bottom panel) datasets, relative abundance of the four taxa enriched in ESCA overall were analyzed by histological subtype, esophageal adenocarcinoma or sequamous cell carcinoma. Significance was determined by Kruskal-Wallis test n.s. not significant, * $p < 0.05$, ** $p < 0.01$, *** $p < 0.001$.

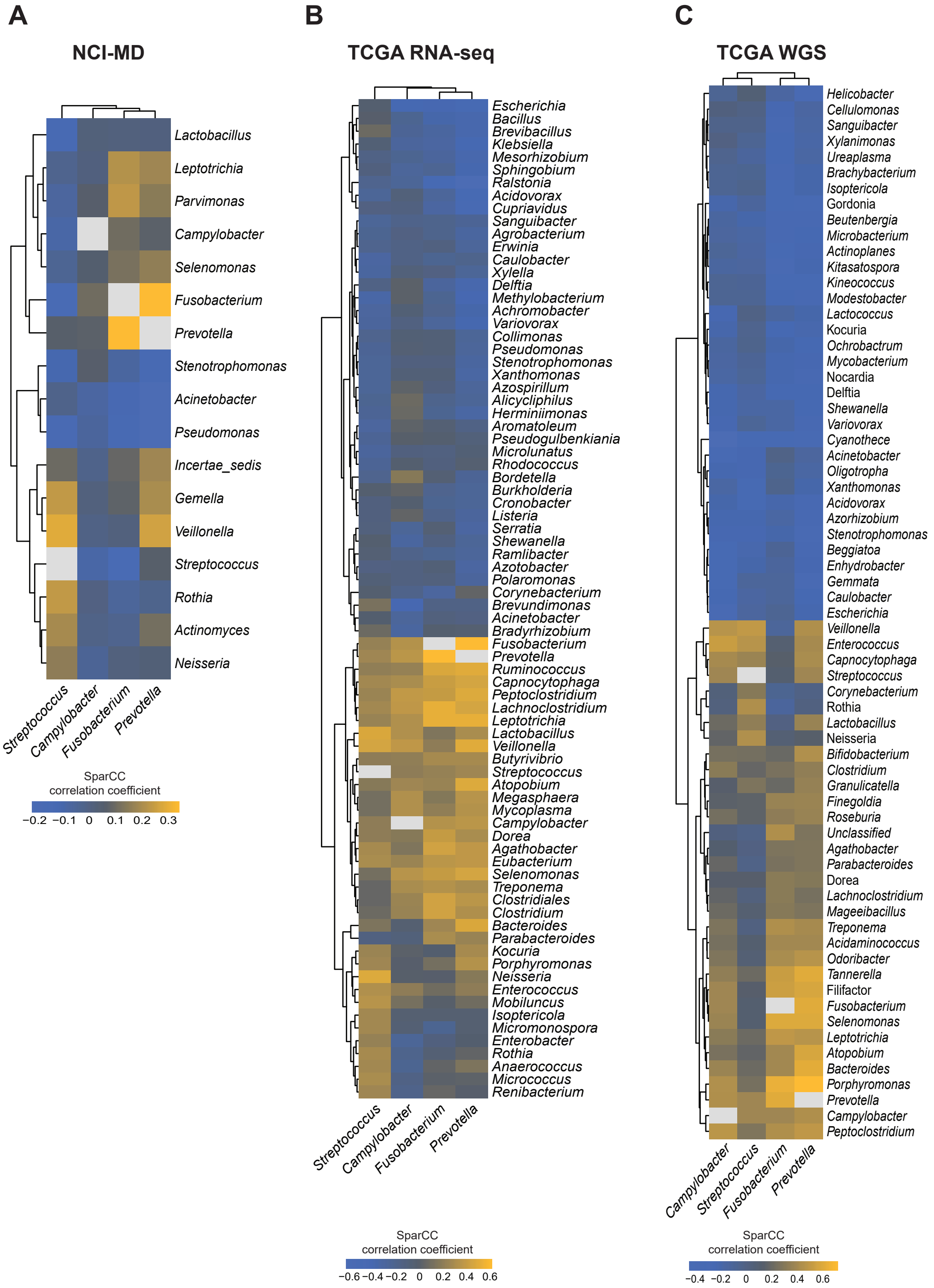

Fig. S5: Heatmap of taxa co-enrichment networks as determined by SparCC. A Taxa enrichment heatmap for NCI-MD case control study with underlying data for network depicted in Fig. 3A. **B** Taxa enrichment heatmap for TCGA RNA-seq with underlying data for network depicted in Fig. 3B. **C** Taxa enrichment heatmap for TCGA WGS with underlying data for network depicted in Fig. 3C. Data was calculated for each heatmap as described in Fig. 3.

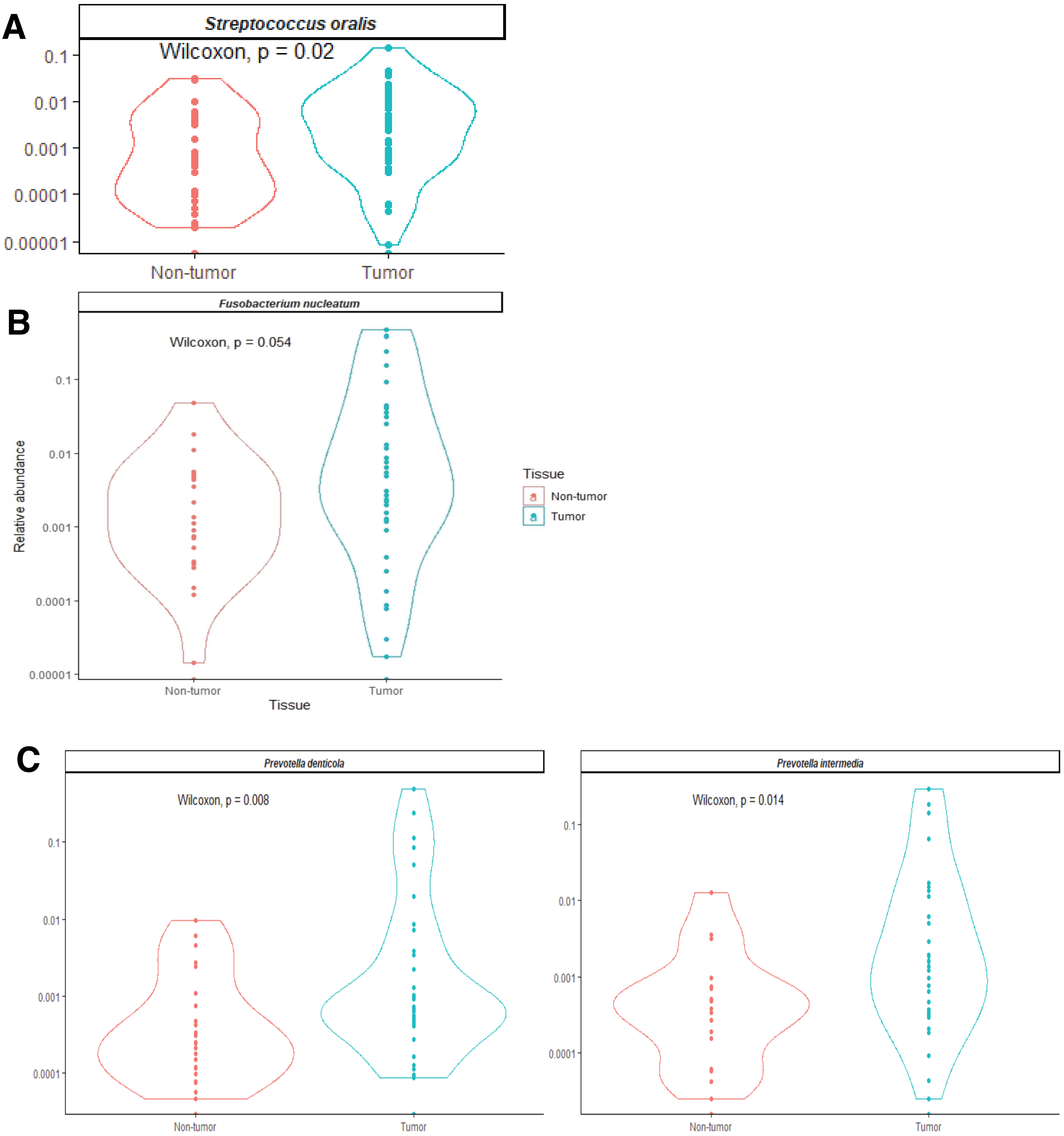

Figure S6. Speciation analysis of co-enriched taxa in EAC tumors and non-tumor. TCGA WGS data were used to obtain species-level data for A) Streptococcus, B) Fusobacterium, and C) Prevotella . Campylobacter did not demonstrate significant enrichment for any species identified (data not shown). *Statistical analysis for difference between relative abundance of species enrichment was calculated using the Wilcoxon test; significant was set at $p<0.05$.

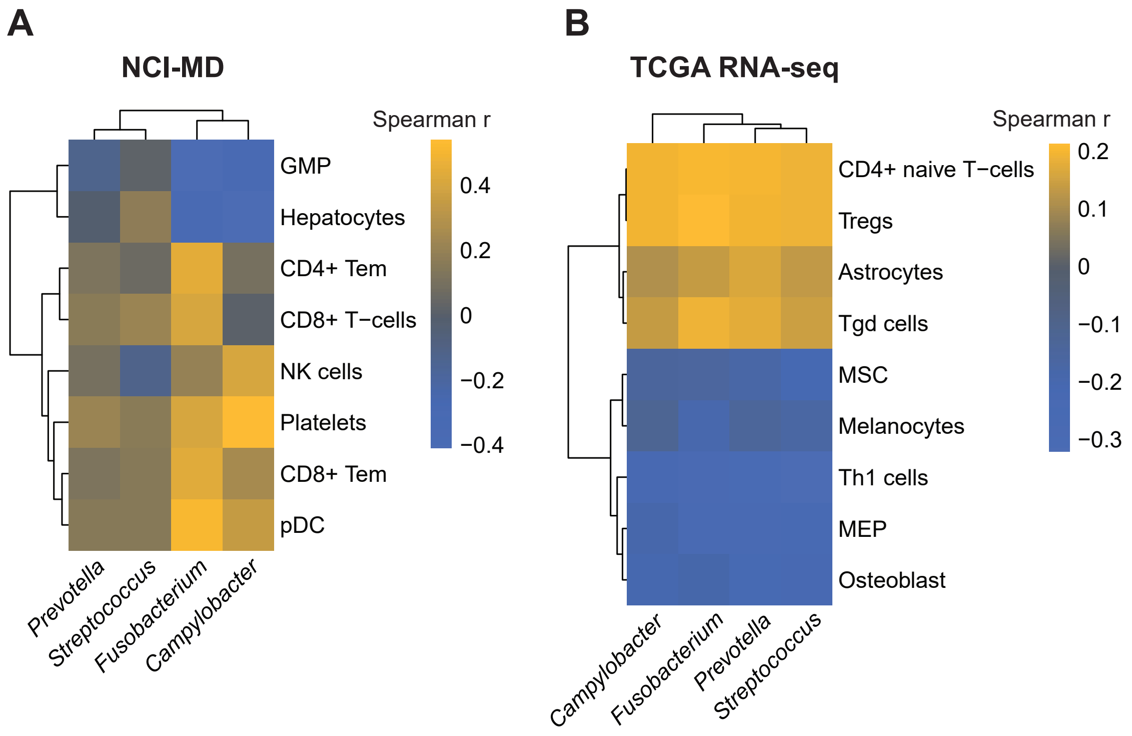

Fig. S7: Heatmap of immune cells significantly enriched or depleted in tumors with high carriage of ESCA-enriched taxa.
A RNA-sequencing was performed on NCI-MD patients (n = 27; non-tumor = 13, BO = 4, tumor = 10) and samples were analyzed for predicted cell infiltration using xCell (citation). Cell infiltrates and taxa abundance were correlated using Spearman's coefficient. **B** Correlation of xCell predicted cell infiltration in TCGA RNA-seq patients with taxa abundance. All correlations shown were sig. $p < 0.05$.

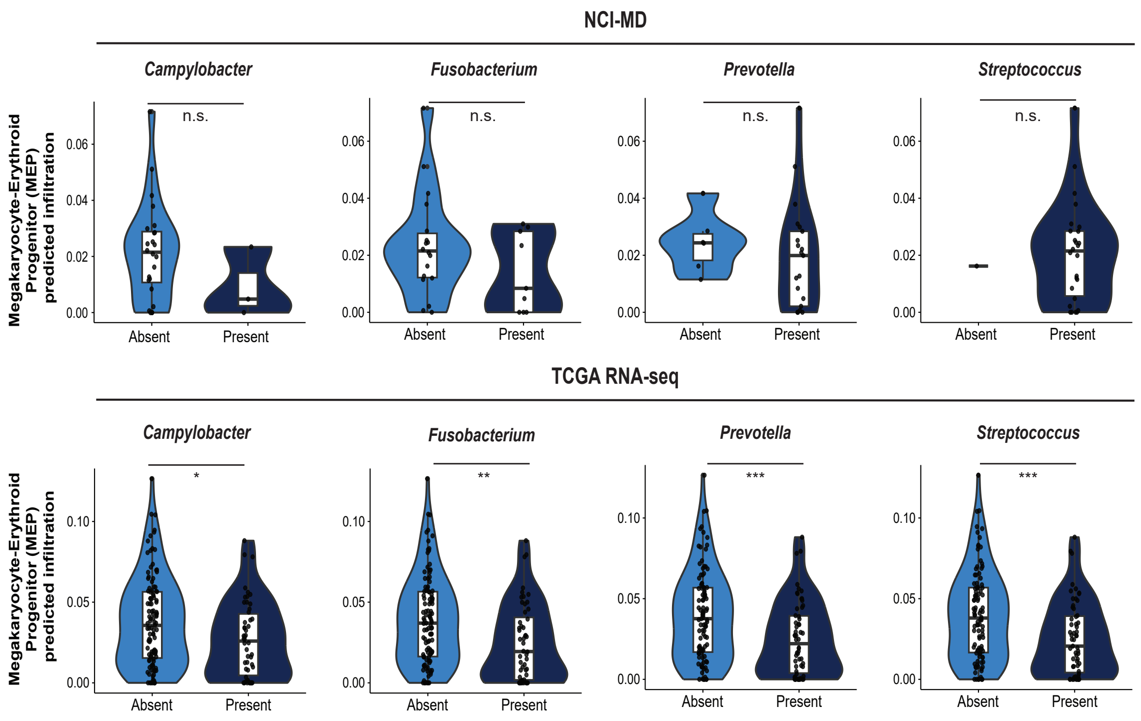

Fig. S8: MEP infiltration is lower in taxa-positive tumors.

Quantification of predicted megakaryocyte-erythroid progenitor (MEP) cell infiltration in the tumor tissues of NCI-MD case control study (top) and TCGA RNA-seq (bottom) present or absent for the indicated taxa. Tumors classified as “present” had one or more reads for the given taxa. MEP infiltration predicted by xCell as described in Fig. 4. Significance determined by Wilcoxon test, n.s. not significant, * $p < 0.05$, ** $p < 0.01$, *** $p < 0.001$.

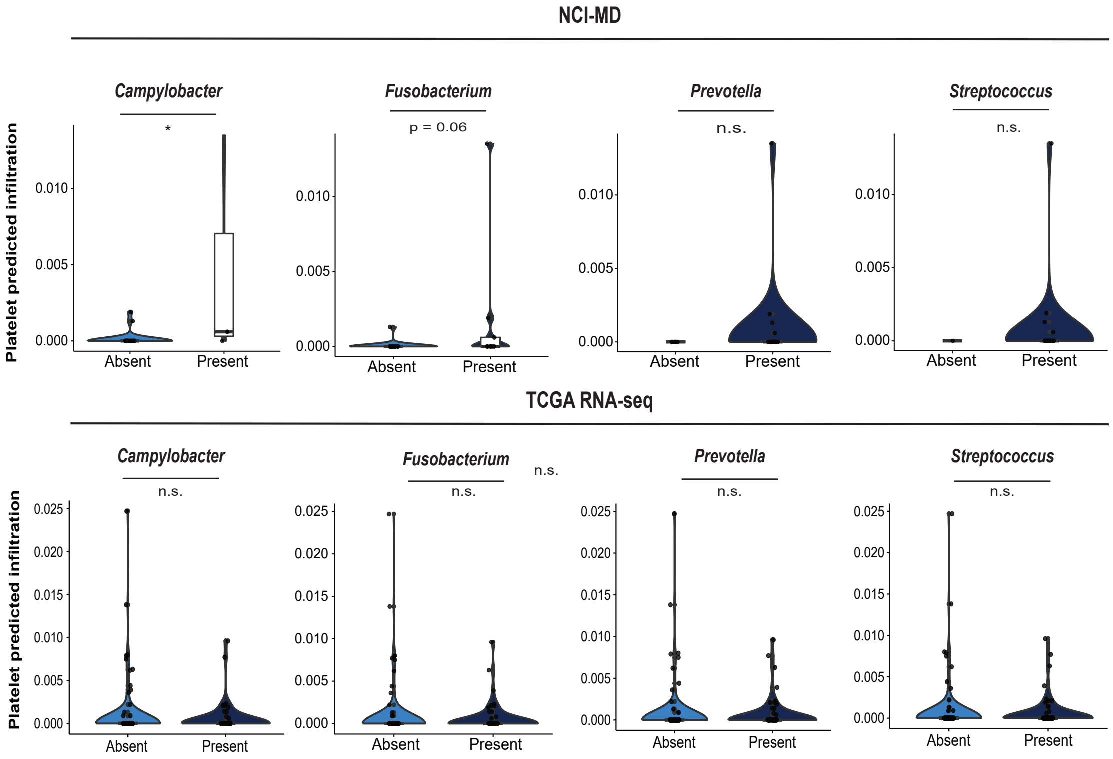

Fig. S9: Platelet infiltration trends higher in taxa-positive tumors.
Quantification of predicted platelet infiltration in the tumor tissues of NCI-MD case control study (top) and TCGA RNA-seq (bottom) present or absent for the indicated taxa. Tumors classified as "present" had one or more reads for the given taxa. Platelet infiltration predicted by xCell as described in Fig. 4. Significance determined by Wilcoxon test, n.s. not significant, * $p < 0.05$.

Primary Cohort (NCI-MD Samples)						
**Description**	**Esophageal adeno-carcinoma tissues**	**Esophageal squamous cell carcinoma tissue**	**Esophageal adeno-carcinoma-adjacent tissues**	**Esophageal squamous cell carcinoma-adjacent tissue**	**Barrett's Esophagus tissue**	**Total**
**N samples**	74	17	87	27	8	213
**Sex (N, %)**						
Male	65 (87.8)	9 (52.9)	77 (88.5)	14 (51.9)	8 (100)	173 (81.2)
Female	9 (12.2)	8 (47.1)	10 (11.5)	13 (48.1)	0 (0.0)	40 (18.8)
**Race (N, %)**						
European American	72 (97.3)	10 (58.8)	85 (97.7)	16 (59.3)	8 (100)	191 (89.7)
African American	1 (1.4)	6 (35.3)	1 (1.1)	9 (33.3)	0 (0.0)	17 (8.0)
Other	1 (1.4)	1 (5.9)	1 (1.1)	2 (7.4)	0 (0.0)	5 (2.3)
**Age, mean ± SD**	60.3 +/- 10.6	60.7 +/- 9.3	61.9 +/- 11.0	58.7 +/- 9.2	65.9 +/- 6.0	61.0 +/- 10.4
**BMI (N, %)**						
underweight	1 (1.4)	2 (11.8)	1 (1.1)	5 (18.5)	0 (0.0)	9 (4.2)
normal weight	21 (28.4)	9 (52.9)	19 (21.8)	11 (40.7)	1 (12.5)	61 (28.6)
overweight	24 (32.4)	5 (29.4)	27 (31.0)	6 (22.2)	2 (25.0)	64 (30.0)
obese	23 (31.1)	1 (5.9)	27 (31.0)	2 (7.4)	3 (37.5)	56 (26.3)
unknown	5 (6.8)	0 (0.0)	13 (14.9)	3 (11.1)	2 (25.0)	23 (10.8)
**History of Barrett's Esophagus (N, %)**						
yes	50 (67.6)	3 (17.6)	60 (69.0)	4 (14.8)	8 (100)	125 (58.7)
no	24 (32.4)	14 (82.4)	27 (31.0)	23 (85.2)	0 (0.0)	88 (41.3)
**History of smoking (N,%)**						
yes	56 (75.7)	14 (82.4)	64 (73.6)	22 (81.5)	5 (62.5)	161 (75.6)
no	15 (20.3)	2 (11.8)	14 (16.1)	2 (7.4)	2 (25.0)	35 (16.4)
missing	3 (4.1)	1 (5.9)	9 (10.3)	3 (11.1)	1 (12.5)	17 (8.0)
**Clinical Stage (N, %)**						
0	2 (2.7)	0 (0.0)	3 (3.4)	0 (0.0)		
I	35 (47.3)	1 (5.9)	12 (13.8)	1 (3.7)		

II	5 (6.8)	6 (35.3)	36 (41.4)	12 (44.4)
III	26 (35.1)	1 (5.9)	28 (32.2)	12 (44.4)
IV	5 (6.8)	9 (52.9)	7 (8.0)	2 (7.4)
missing	0 (0.0)	0 (0.0)	0 (0.0)	0 (0.0)
**Neoadjuvant therapy (N, %)**	44 (59.5)	9 (52.9)	52 (59.8)	18 (66.7)
**Survival (days), mean ± SD**	1922.9 +/- 1813.0	1924.0 +/- 1872.0	1848.7 +/- 1754.4	1699 .2+/- 1911.4

Table S1. NCIMD case control cohort study sample demographics

Validation Cohort (The Cancer Genome Atlas – RNA-seq Samples)					
**Description**	**Esophageal adeno-carcinoma tissues**	**Esophageal squamous cell carcinoma tissue**	**Esophageal adeno-carcinoma-adjacent tissues**	**Esophageal squamous cell carcinoma-adjacent tissue**	**Total**
**N samples**	28	31	7	0	66
**Sex (N, %)**					
Male	22 (78.6)	28 (90.3)	5 (71.4)	0 (0.0)	55 (83.3)
Female	6 (21.4)	3 (9.7)	2 (28.6)	0 (0.0)	11 (16.7)
**Race (N, %)**					
European American	23 (82.1)	10 (32.3)	7 (100.0)	0 (0.0)	40 (60.6)
African American	0 (0.0)	1 (3.2)	0 (0.0)	0 (0.0)	1 (1.5)
Asian American	1 (3.6)	20 (64.5)	0 (0.0)	0 (0.0)	21 (31.8)
Other	4 (14.3)	0 (0.0)	0 (0.0)	0 (0.0)	4 (6.1)
**Age, mean ± SD**	67.2 +/- 13.2	58.6 +/- 11.1	75.7 +/- 6.9	N/A	64.1 +/- 12.9
**BMI (N, %)**					
underweight	1 (3.6)	3 (9.7)	0 (0.0)	0 (0.0)	4 (6.1)
normal weight	7 (25.0)	23 (74.2)	1 (14.3)	0 (0.0)	31 (47.0)
overweight	8 (28.6)	2 (6.5)	5 (71.4)	0 (0.0)	11 (16.7)
obese	11 (39.3)	1 (3.2)	1 (14.3)	0 (0.0)	17 (25.8)
unknown	1 (3.6)	2 (6.5)	0 (0.0)	0 (0.0)	3 (4.6)
**History of Barrett's Esophagus (N, %)**					
yes	7 (25.0)	0 (0.0)	2 (28.6)	0 (0.0)	9 (13.6)
no	17 (60.7)	13 (41.9)	5 (71.4)	0 (0.0)	35 (53.0)

missing	4 (14.3)	18 (58.1)	0 (0.0)	0 (0.0)	22 (33.3)
**History of smoking (N,%)**					
yes	19 (67.9)	17 (54.8)	4 (57.1)	0 (0.0)	40 (60.6)
no	9 (32.1)	14 (0.452)	3 (42.9)	0 (0.0)	26 (39.4)
**Clinical Stage (N, %)**					
0	0 (0.0	0 (0.0)	0 (0.0)	0 (0.0)	
I	6 (21.3)	3 (9.7)	3 (42.9)	0 (0.0)	
II	7 (25.0)	18 (58.0)	1 (14.3)	0 (0.0)	
III	7 (25.0)	8 (25.8)	2 (28.6)	0 (0.0)	
IV	1 (3.6)	1 (3.2)	0 (0.0)	0 (0.0)	
missing	7 (25.0)	1 (3.2)	1 (14.3)	0 (0.0)	
**Survival (days), mean ± SD**	444.0 +/- 465.0	452.0 +/- 432.0	313.0 +/- 423.0	N/A	

Table S2. TCGA RNA-seq cohort sample demographics

Validation Cohort (The Cancer Genome Atlas – Whole Genome Sequenced Samples)					
**Description**	**Esophageal adeno-carcinoma tissues**	**Esophageal squamous cell carcinoma tissue**	**Esophageal adeno-carcinoma-adjacent tissues**	**Esophageal squamous cell carcinoma-adjacent tissue**	**Total**
**N samples**	15	29	16	35	95
**Sex (N, %)**					
Male	11 (73.3)	25 (86.2)	11 (68.8)	30 (85.7)	77 (81.1)
Female	4 (26.7)	4 (13.8)	5 (31.2)	5 (14.3)	18 (18.9)
**Race (N, %)**					
European American	14 (93.3)	12 (41.4)	16 (100.0)	13 (37.1)	55 (57.9)
African American	0 (0.0)	1 (3.5)	0 (0.0)	2 (5.7)	3 (3.2)
Asian American	1 (6.7)	16 (55.2)	0 (0.0)	20 (57.1)	37 (38.9)
Other	0 (0.0)	0 (0.0)	0 (0.0)	0 (0.0)	0 (0.0)
**Age, mean ± SD**	70.8 +/- 11.2	60.2 +/- 11.0	73.3 +/-8.5	59.6 +/- 11.3	63.9 +/- 12.1
**BMI (N, %)**					
underweight	0 (0.0)	3 (10.3)	0 (0.0)	4 (11.4)	7 (7.4)
normal weight	4 (26.7)	18 (62.1)	4 (25.0)	23 (65.7)	49 (51.6)
overweight	2 (13.3)	5 (17.2)	2 (12.5)	5 (14.3)	14 (14.7)
obese	8 (53.3)	1 (3.5)	9 (56.2)	1 (2.9)	19 (20.0)
unknown	1 (6.7)	2 (6.9)	1 (6.3)	2 (5.7)	6 (6.3)

**History of Barrett's Esophagus (N, %)**					
yes	4 (26.7)	0 (0.0)	5 (31.2)	0 (0.0)	9 (9.5)
no	8 (53.3)	13 (44.8)	8 (50.0)	16 (45.7)	45 (47.4)
missing	3 (20.0)	16 (55.2)	3 (18.8)	19 (54.3)	41 (43.2)
**History of smoking (N,%)**					
yes	9 (60.0)	14 (48.3)	11 (68.8)	15 (42.9)	49 (51.6)
no	6 (40.0)	15 (51.7)	5 (31.2)	20 (57.1)	46 (48.4)
**Clinical Stage (N, %)**					
0	0 (0.0)	0 (0.0)	0 (0.0)	0 (0.0)	
I	5 (33.3)	2 (6.9)	7 (43.8)	2 (5.7)	
II	4 (26.7)	15 (51.7)	4 (25.0)	20 (57.1)	
III	3 (20.0)	9 (31.0)	2 (12.5)	9 (25.7)	
IV	1 (6.7)	2 (6.9)	1 (6.3)	2 (5.7)	
missing	2 (13.3)	1 (3.4)	2 (12.5)	2 (5.7)	
**Survival (days), mean ± SD**	608.0 +/- 275.0	397.0 +/- 501.0	560.0 +/- 292.0	361.0 +/- 475.0	

Table S3. TCGA RNA-seq cohort sample demographics

	Mean % Abundance in Tumor Samples		
Species	16S rRNA	WGS	RNAseq
*Streptococcus spp.**	20.17	15.52	1.11
Prevotella melaninogenica	1.99	10.25	0.34
Fusobacterium nucleatum	4.38	6.31	2.07
Veillonella parvula	1.34	4.61	0.42
Helicobacter pylori	0.18	3.23	0.01
Haemophilus influenzae	0.22	2.86	0.66
Prevotella denticola	0.13	2.22	0.17
Prevotella intermedia	0.80	1.81	0.05
Campylobacter concisus	0.14	1.60	0.03
Staphylococcus aureus	0.54	1.23	0.16
Rothia mucilaginosa	3.16	1.09	0.54
Haemophilus parainfluenzae	1.74	1.09	0.09
Selenomonas sputigena	1.96	1.01	0.08
Bacteroides fragilis	0.16	0.77	0.03
Stenotrophomonas maltophilia	3.88	0.47	0.41
Gemella haemolysans	1.55	0.32	0.16

S. oralis, S. mitis, S. pneumoniae, S. parasanguinis, S. salivarius

		Comparative Statistical Results					Statistical Frequency Results		
		GLM P-value	GLMM P-value	Mann-Whitney Tumor vs Non-Tumor			Fisher's Exact Test for Tumor vs Normal		
Species	Cohort Exclusion	Tumor vs Non-tumor	Tumor vs Non-tumor	16SrRNA	WGS	RNAseq	16SrRNA	WGS	RNAseq
Fusobacterium nucleatum	No WGS Blood	0.0870	0.0054	0.0000	0.4602	0.0524	0.0004	0.8134	0.0471
Fusobacterium nucleatum	WGS Non-tumor w/ Blood	0.0015	0.0001	0.0000	0.0074	0.0524	0.0004	0.0253	0.0471

		Comparative Statistical Results					Statistical Frequency Results		
		GLM P-value	GLMM P-value	Mann-Whitney Tumor vs Non-Tumor			Fisher's Exact Test for Tumor vs Normal		
Species	Cohort Exclusion	Tumor vs Non-tumor	Tumor vs Non-tumor	16SrRNA	WGS	RNAseq	16SrRNA	WGS	RNAseq
Streptococcus spp	No WGS Blood	0.3952	0.5795	0.0154	0.6974	0.2194	0.0228	0.7872	0.1066
Streptococcus spp	WGS Non-tumor w/ Blood	0.0873	0.0151	0.0154	0.0346	0.2194	0.0228	0.0127	0.1066

		Comparative Statistical Results					Statistical Frequency Results		
		GLM P-value	GLMM P-value	Mann-Whitney Tumor vs Non-Tumor			Fisher's Exact Test for Tumor vs Normal		
Species	Cohort Exclusion	Tumor vs Non-tumor	Tumor vs Non-tumor	16SrRNA	WGS	RNAseq	16SrRNA	WGS	RNAseq
Campylobacter concisus	No WGS Blood	0.2805	0.4084	0.0047	0.6204	0.0388	0.0045	1.0000	0.0419
Campylobacter concisus	WGS Non-tumor w/ Blood	0.2148	0.1485	0.0047	0.0077	0.0388	0.0045	0.0089	0.0419

		Comparative Statistical Results					Statistical Frequency Results		
		GLM P-value	GLMM P-value	Mann-Whitney Tumor vs Non-Tumor			Fisher's Exact Test for Tumor vs Normal		
Species	Cohort Exclusion	Tumor vs Non-tumor	Tumor vs Non-tumor	16SrRNA	WGS	RNAseq	16SrRNA	WGS	RNAseq
Prevotella melaninogenica	No WGS Blood	0.9021	0.8310	0.9459	0.1724	0.0797	1.0000	0.1254	0.1884
Prevotella melaninogenica	WGS Non-tumor w/ Blood	0.0002	0.0006	0.9459	0.0000	0.0797	1.0000	0.0000	0.1884

Table S4. Mean relative abundance across cohorts sorted first by species in WGS and comparative statical comparisons of four taxa enriched in tumors vs non-tumor adjacent.

	Campylobacter			*Fusobacterium*			*Prevotella*			*Streptococcus*		
**Genus**	**NCIMD**	**RNA**	**WGS**	**NCIMD**	**RNA**	**WGS**	**NCIMD**	**RNA**	**WGS**	**NCIMD**	**RNA**	**WGS**
Campylobacter	NA	NA	NA	0.0977	0.3314	0.3733	0.0692	0.3494	0.4239	-0.0923	0.1915	0.3829
Fusobacterium	0.0977	0.3314	0.3733	NA	NA	NA	0.3371	0.609	0.6038	-0.2083	0.2132	0.0937
Leptotrichia	0.0072	0.3211	0.3086	0.2125	0.5202	0.4781	0.1792	0.5202	0.4643	-0.0326	0.2232	0.2
Neisseria	-0.0485	-0.0019		0.0138	-0.0152		0.0233	0.1697		0.1532	0.4782	
Prevotella	0.0692	0.3494	0.4239	0.3371	0.609	0.6038	NA	NA	NA	0.0637	0.2522	0.3786
Selenomonas	0.0531		0.3642	0.1159		0.5826	0.1559		0.5889	-0.0460		0.0997
Streptococcus	-0.0923	0.1915	0.3829	-0.2083	0.2132	0.0937	0.0637	0.2522	0.3786	NA	NA	NA
Veillonella	-0.0134	0.3612	0.4588	0.0137	0.1892	-0.123	0.2569	0.4912	0.4286	0.2815	0.4452	0.4925

Table S5. Concordance between taxonomic co-occurrence and co-exclusion
